# Ontogenetic Changes in Blood Osmolality During the Postembryonic Development of Zebrafish (*Danio rerio*)

**DOI:** 10.1089/zeb.2021.0075

**Published:** 2022-02-14

**Authors:** Guy Charmantier, Mai Nguyen-Chi, Georges Lutfalla

**Affiliations:** ^1^ CNRS, Ifremer, IRD, UM, Marbec, University of Montpellier, Montpellier, France.; ^2^ LPHI, CNRS, University of Montpellier, Montpellier, France.

**Keywords:** teleost, zebrafish, ontogeny, osmoregulation, nano-osmometry

## Abstract

The zebrafish *Danio rerio* is a teleost model species widely used in developmental genetics, biomedical studies, toxicology, and drug screening. Despite the interest of this species in research, little is known through indirect observations about its blood osmolality, which is a key parameter for diverse experiments. In this study, we directly measured blood osmolality using nano-osmometry at different stages of zebrafish postembryonic development. We found that blood osmolality is close to 240 mOsm·kg^−1^ in early larvae. It progressively increased to ∼270 mOsm·kg^−1^ during the larval development before reaching ∼300 mOsm·kg^−1^ after metamorphosis in juveniles and later in adults. These ontogenetic changes in blood osmolality illustrate the physiological changes in osmoregulation associated with postembryonic development, including metamorphosis. These values are of practical interest for adjusting the osmolality of fixatives and cell and tissue culture media for research using zebrafish as a model.

## Introduction

The zebrafish *Danio rerio* (Buchanan-Hamilton 1822) is a stenohaline freshwater teleost (Cyprinidae). Its main natural range covers the Ganges and Brahmaputra River basins in India, Bangladesh, and Myanmar where it is typically found in slow-moving or standing water bodies.^1^ Exported over a century ago, it became a common aquarium species easy to culture. This feature and other traits such as small size, rapid development, optical transparency of early embryos, availability of genetic databases, and applicability of molecular tools, have made the zebrafish a key model for developmental genetics, toxicology, drug screening, and biomedical studies, including cancer, host–pathogen interactions and regenerative medicine. The experimental use of this fish has been facilitated by descriptions of its embryonic^2^ and postembryonic^3^ stages of development, and of practical rules for its culture.^4^

Embryos are able to develop below 4‰ (∼110 mOsm·kg^−1^),^5^ and embryos and early larvae can stand extremely low ion concentrations of 34 μM Na^+^, 40 μM Cl^−^ (salinity ∼0.003‰; osmolality ∼0.08 mOsm·kg^−1^).^6^ In later stages of development, it has been shown that adult zebrafish can regulate plasma and whole-body electrolyte concentrations,^7^ from soft waters with low ion concentrations (Na^+^: 35 μM; Cl^−^: 43 μM) to hard water (Na^+^: 1480 μM; Cl^−^: 1625 μM), with corresponding osmolalities of 0.1 to 7–10 mOsm·kg^−1^. Acute exposures to higher salinity have been reported as tolerable in adults (without indication of the duration of exposure) up to 20–25‰, with “highly disrupted epithelium” of the gills at the latter salinity.^8^

In freshwater, the zebrafish is submitted to a passive loss of ions, compensated by ion uptake from the external medium and limited by production of dilute urine, resulting in the regulation of the osmolality of the blood as in other freshwater teleosts.^9^ *D. rerio* has become a model for the study of epithelial transport associated with ionic and osmotic regulation. The ion-transporting cells or ionocytes, their structure, and sequential development from skin in embryos to gills in postembryonic stages, their different types, and their differentiation pathways, have been studied in embryos,^10–14^ in larvae,^10,11,15^ and in adults.^10,16–19^ Ionocytes are involved in ion transport, particularly in ion uptake from ion-poor media. Their functions, including endocrine control and gene expression, have been studied in adults^7,20^ and in early developmental stages.^11,17–19^

Despite this wide interest over the past twenty years regarding the ionocyte structure and function in zebrafish, and despite the importance of this fish as a key species in research, little is known about the value of its blood osmolality, although it represents a basic parameter for diverse experiments. Very few data obtained through indirect observations are available on the actual level of the blood osmolality at each developmental stage. In addition to their scientific interest, knowing these values would yet be valuable from a practical point of view, for instance to precisely adjust the osmolality of fixatives and of media used in organ and cell cultures.

Blood osmolality of zebrafish is generally estimated from the average blood osmolality of other adult freshwater teleosts, which is in the range 260–320 mOsm·kg^−1^ according to species^9^; and “most authors assume that adults and larvae share the same osmolality of body fluids.”^21^ Consequently, the estimated blood osmolality in early developmental stages of *D. rerio* has been deemed either equal to that in adults, close to 300–320 mOsm·kg^−1^ or lower (Table 1).^22–27^

**Table 1.** Blood Osmolality (mOsm·kg^−^^1^) Evaluated by Different Authors in *Danio Rerio* at Different Stages

Embryo	Early develop.: Larva	Mid develop.: Juvenile	Adult	Method	Reference
Mid-blastula Optimum 315				Culture in different media	^25^
Cell injection recommended 300				Assumption	^26^
	Same osmolality	Same osmolality		Assumption	^22^
	Lower osmolality	Lower osmolality		Assumption	^23^
	Lower osmolality	Lower osmolality		Assumption	^24^
	Same osmolality	Same osmolality		Assumption	^26^
	Same osmolality	Same osmolality		Assumption	^27^
			Na^+^ & Cl^−^: 210 mM (soft water) 260 mM (hard water)	Plasma, ion chromatography	^7^
	4–5 dpf 240			Test on crystalline lenses at 180, 240, & 320 mOsm·kg^−1^	^21^
			320	Estimated from other studies	^21^

Soft water: 48 μM Na^+^ & Cl^−^; 4 μM Ca^++^. Hard water: 3100 μM Na^+^ & Cl^−^; 3200 μM Ca^++^. “Same” and “lower” osmolality: compared with adults in which blood osmolality is generally estimated at 320 mOsm·kg^−1^.

dpf, days postfertilization.

To our knowledge, only two studies provide preliminary data on ionic concentration or osmolality of the zebrafish blood. One of them reports that total plasma Na^+^ and Cl^−^ concentrations in adult zebrafish were 210 and 260 mM, respectively, in soft and hard water (0.08 and 3 mOsm·kg^−1^) (Table 1).^7^ The second study was based on an indirect method, the microscopic observation of the shape and deformations of freshly excised lenses from zebrafish eyes at 5 days postfertilization (dpf) immersed in three media at different salinities, 180, 240, and 320 mOsm·kg^−1^.^21^ Only in the 240 mOsm·kg^−1^ was the eye structure preserved. The authors concluded that the osmotic concentration of body fluids of the zebrafish was ∼240 mOsm·kg^−1^ in 5-dpf-old larvae, lower than the “established adult value of 320 mOsm·kg^−1^” (Table 1).^21^

However, it has been known for a long time from studies conducted in several teleost species that the capacity to osmoregulate undergoes ontogenetic modifications: it generally changes at selected times such as hatching and metamorphosis, resulting in increased blood osmolality during development.^28–30^

This review of the available published studies shows that, in contrast to other teleost species, there is a lack of precise data on blood osmolality values at different stages of the zebrafish. This probably originates from technical limitations in—properly sampling blood from tiny animals, and—reliably measuring osmolality on minute volumes of blood.

As the results reported above for *D. rerio* are scarce in a limited number of stages, the objective of the present study was to directly measure the values of blood osmolality during the entire postembryonic development of the zebrafish, using nano-osmometry that allowed to collect individual data in all stages but the smallest one tested.

## Materials and Methods

### Animals

Specimens of *D. rerio* were obtained from the culture facility of the University of Montpellier. For the present studies, we used the wild-type reference AB line (https://zfin.org/ZDB-GENO-960809-7).

In the laboratory, females were kept in 3.5 L polycarbonate aquaria connected to a closed recirculating system (Tecniplast), in which fresh water with addition of Instant Ocean salts at 0.06 g·L^−1^ was adjusted at pH close to 7 by the addition of 40 μL of NaOH 10 N per liter. Constant conditions in the laboratory were 0.4‰ salinity/4 mOsm·kg^−1^ osmolality/400 μS·cm^−1^ conductivity, a temperature of 28°C and a 12-h light:12-h dark cycle. The fish were fed twice per day. Newly hatched larvae were collected in Petri dishes and reared in glass vials (∼300 mL) under the same conditions concerning water, temperature, and light. Larvae were fed from days 5.

Before experiments, the weight and length (SL [standard length], from snout to caudal peduncle^3^) of 3-week-old and older individuals were measured. For staging the animals during their postembryonic life, we used the criteria based on externally visible anatomy according to Parichy *et al.*^3^ Following the hatching period at 48–72 hpf (hours postfertilization),^2,3^ larvae develop until becoming juveniles following a metamorphic process.

During metamorphosis, the morphology, anatomy, and physiology of the larvae change, with loss or remodeling of larval features and acquisition of juvenile and adult features. Juveniles have most adult features in the absence of sexual maturity, with corresponding changes in behavior and ecology in the wild.^3^ In the culture conditions used in this study, the metamorphic transition between larva and juvenile occurred at ∼6 weeks pf and the transition from juvenile to adult occurred at ∼2–3 months pf.

The developmental stages tested in the osmoregulation experiment included stages from early larvae to adults. We conducted individual measurements of blood osmolality at the following stages: 3-week-old larvae (L 3wk), 6-week-old larvae/juveniles (L-Juv 6wk), 2-month-old and 3-month-old juveniles/adults (Juv-Ad 2m, Juv-Ad 3m), and 2-year-old and 2-year-1-month-old adults (Ad 2yr, Ad 2yr1m). As early larvae were too small for individual sampling, pools of 5-dpf-old larvae (L 5d) were prepared (see below).

### Measurement of blood osmolality

All experiments were conducted according to the European Union guidelines for handling of laboratory animals (http://ec.europa.eu/environment/chemicals/lab_animals/home_en.htm) and were approved by the Comité d'Éthique pour l'Expérimentation Animale under reference CEEA-LR- B4-172-37 and APAFIS#5737-2016061511212601 v3.

Before sampling, all individuals were anesthetized in fish water supplemented with 0.016% tricaine.

The weight and length (SL, from snout to caudal peduncle^3^) of individuals were measured from 3-week-old larvae (5.1 mm < SL <6.4 mm) and in later stages. For the measure of blood osmolality, we used a cryoscopic method on a nano-osmometer requiring small volumes of about 30 nL, which has been successfully applied in several small-sized animals such as crustacean and fish larvae.^30,31^ Each animal was carefully dried on filter paper, and then quickly immersed in mineral oil to prevent evaporation and desiccation. Blood was sampled with a hand-made glass micropipette inserted into the heart. Blood osmolality was measured with reference to a 300 mOsm·kg^−1^ standard solution (Löser-Messtechnik, Berlin) on a Kalber–Clifton nanoliter osmometer (Clifton Technical Physics, Hartford, NY).

In 5-day-old larvae, preliminary attempts revealed that blood sampling was made difficult and unreliable by their small size (3.3 mm < SL <3.9 mm). To evaluate the osmolality of their body fluids, pools of 300–500 larvae were used. After being carefully spread and quickly dried on filter paper, they were ground in a potter and the resulting suspension was centrifuged for 2 min at 19000 G. The supernatant was then sampled and transferred to the nanoliter osmometer for osmolality measurement.

### Statistical analysis

Groups include the number of independent values, and statistical analysis was done using these independent values. GraphPad Prism 7 Software (San Diego, CA) was used to construct graph and analyze data. Kruskal–Wallis one-way analysis of variance (ANOVA) test with Dunn's multiple comparisons post-test were used to evaluate the significance of the differences between groups.

## Results

Blood osmolality values (mean ± standard deviation) in adult males (302.4 ± 8.25 mOmsm·kg^−1^, *n* = 11) and females (303.2 ± 9.96 mOsm·kg^−1^, *n* = 5) were not significantly different (Mann–Whitney test, two-tailed, *p* = 0.9789); therefore, values from both sexes were pooled.

**FIG. 1.**
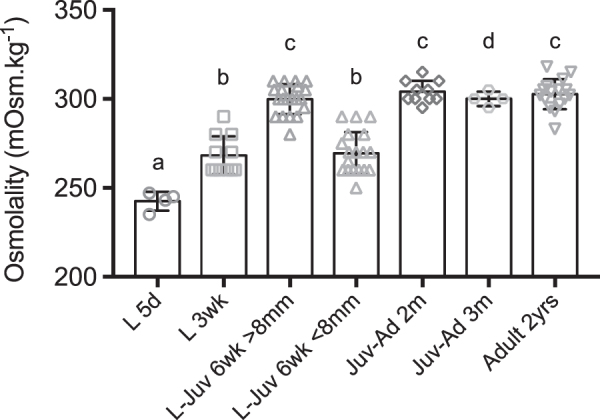
Ontogeny of osmoregulation of *Danio rerio*, in fresh water at 28°C. The graph represents the values of blood osmolality at different stages of postembryonic development. Dots show values measured on pooled larvae (L 5d) and individual values (from L 3wk to Ad). Columns show mean ± SD. Different letters indicate significant differences. a: significantly different (*p* < 0.05, *p* < 0.01, *p* < 0.005) from L-Juv 6wk >8 mm, Juv-Ad 2m, Juv-Ad 3m, Ad 2yrs. b: significantly different (*p* < 0.05, *p* < 0.01, *p* < 0.005) from L-Juv 6wk >8 mm, Juv-Ad 2m, Ad 2yrs. c: significantly different (*p* < 0.01, *p* < 0.005) from L 5d, L 3wk, L-Juv 6wk <8 mm. d: significantly different (*p* < 0.05) from L 5d. L: Larva; Juv: Juvenile; Ad: Adult; d: day; wk: week; m: month; yr: year.

Blood osmolality increased during the postembryonic development from larvae to juveniles and adults of *D. rerio* (Table 2 and Fig. 1). The lowest value was found in body fluids from homogenates of 5-dpf-old larvae, at 243 mOsm·kg^−1^. Mean blood osmolality was 268 mOsm·kg^−1^ in 3-week-old larvae and 285 mOsm·kg^−1^ in a group of 6-week-old larvae/juveniles. In the latter, two subgroups were distinguished according to their size (SL): the mean blood osmolality was 270 mOsm·kg^−1^ in the smaller larvae (SL: 7.4 ± 0.2 mm), not different from the value in 3-week-old larvae (*p* > 0.9999), but in the bigger 6-week-old larvae-juveniles (SL: 10.6 ± 1.3 mm), the mean blood osmolality was 300 mOsm·kg^−1^, higher than in 3-week-old larvae (*p* < 0.001) (Table 2 and Supplementary Table S1).

**Table 2.** Ontogeny of Osmoregulation of *Danio rerio*, in Fresh Water at 28°C

Osmolality of FW mOsm·kg^−1^	Stage	Length mm SL Mean ± SD; (*n*)	Length mm Min–max	Weight mg Mean ± SD; (*n*)	Weight mg Min–max	Blood osmolality mOsm·kg^−1^ Mean ± SD; (*n*)	Blood osmolality mOsm·kg^−1^ Min–max
4	**L 5d**	3.6 ± 0.2; (12)	3.3–3.9			**243** (±5) (4 pools of 300–500)^a^	235–247^a^
4	**L 3wk**	5.9 ± 0.3; (11)	5.1–6.4	3.2 ± 0.4; (11)	2.8–3.5	**268 ± 11;** (11)	260–290
4	**L-Juv 6wk**	9.0 ± 2.0; (39)	6.9–12.2	8.1 ± 4.5; (39)	3.2–22.9	**285 ± 18;** (39)	250–310
4	Subgroup SL >8 mm	10.6 ± 1.3; (20)	8.7–12.2	11.2 ± 4.3; (20)	6.0–22.9	**300 ± 8;** (20)	280–310
4	Subgroup. SL <8 mm	7.4 ± 0.2; (19)	6.9–7.8	4.8 ± 1.2; (19)	3.2–7.8	**270 ± 12;** (19)	250–290
4	**Juv-Ad 2m**	18 ± 3; (10)	15–22	100 ± 62; (10)	42–136	**304 ± 6;** (10)	295–315
4	**Juv-Ad 3m**	21 ± 3; (4)	18–24	141 ± 55; (4)	78–193	**300 ± 4;** (4)	295–305
4	**Adult 2yr**	33 ± 3; (10)	30–37			**303 ± 10;** (10)	283–315
4	**Adult 2yr 1m**	33.9 ± 2.3; (6)	31.8–37.5	782.9 ± 317.6; (6) 580.9 ± 46.2; (4m) 1187 ± 89.7; (2f)	531–1250 (6) 531–642 (4m) 1124–1250 (2f)	**302 ± 5;** (6)	295–311

Main stages and the main osmolality values are highlighted in bold.

Values of length, weight, and blood osmolality at different stages of postembryonic development. Mean ± SD, with minimum–maximum range. For the weight results (fifth and sixth columns) in Adult 2yr 1m: *n*, total 6, including 4 males (m) and 2 females (f).

^a^ Osmolality of body fluids.

L: Larva; Juv: Juvenile; Ad: Adult; wk: week; m: month; yr: year; SD, standard deviation.

Later in development, 2- and 3-month-old juvenile-adults had a mean blood osmolality of 300–304 mOsm·kg^−1^, also higher than in 3-week-old larvae (*p* < 0.001 for 2-month-old juvenile-adults vs. 3-week-old larvae). Adult fish, 2-year-old and tested twice, yielded similar mean blood values, 302 and 303 mOsm·kg^−1^ (*p* < 0.001 for 2-year-old vs. 3-week-old larvae, Supplementary Table S1).

## Discussion

A cryoscopic nano-osmometer allows direct measurements of osmolality on very small volumes of liquid down to 30 nL. This technique was successfully used to measure the blood osmolality on individual zebrafish at different developmental stages, from 3-week-old larvae to juveniles and adults.

The blood osmolality of adult zebrafish was close to 300 mOsm·kg^−1^. In a study of ionic regulation in this species,^7^ it was reported that total plasma Na^+^ and Cl^−^ concentration in adult zebrafish was 260 mM (or mEq·L^−1^) in hard water at 3 mOsm·kg^−1^, close to the water used in this study (Table 1). As Na^+^ and Cl^−^ build up ∼90% of the total blood osmolality,^9^ these ion concentrations correspond to an estimated osmolality of ∼290 mOsm·kg^−1^, close to our own finding that is lower than the estimated value of 320 mOsm·kg^−1^ (the “textbook value”) cited in a recent study.^21^ In other adult freshwater teleosts, the measured values of blood osmolality range from ∼260 to ∼330 mOsm·kg^−1^ according to species.^21,27,32,33^

In larval stages, we found through our measurements that blood osmolality was lower than in adults, which is in agreement with previous indirect observations.^21^ Due to the small size of early larvae at 5 dpf preventing reliable sampling of sufficient volume of blood, osmolality measurements were conducted on the pooled body fluids from hundreds of individuals at this stage. The measured value of 243 mOsm·kg^−1^ may be slightly higher than the actual blood osmolality as, in addition to ions, intracellular osmolytes such as small carbohydrates and free amino acids are found in body fluids. This value is however very close to that of 240 mOsm·kg^−1^ indirectly evaluated^21^ from the observation of the shape of the eye lenses immersed in solutions at different osmolalities.

Later in development, in 3-week-old larvae, blood osmolality was higher at 268 mOsm·kg^−1^ and it increased again 3 weeks later in 6-week-old animals. However, at this time of development, the fish size varied between the individuals: in the smaller ones (SL, ∼7 mm), blood osmolality was close to that in 3-week-old larvae (269 mOsm·kg^−1^), but it was significantly higher (300 mOsm·kg^−1^) in the bigger fish (SL ∼10–11 mm).

We consider that the smaller 6-week-old fish were still larvae, with unchanged osmolality compared with 3-week-old larvae, while the bigger 6-week-old fish were juveniles, with higher osmolality originating from metamorphic changes. Under culture conditions used in this study, metamorphosis thus occurred from ∼6 weeks onward for a SL value of 9 to 12 mm (Table 2). In a detailed study of the staging of developing *D. rerio*,^3^ a timing of 4- to 6–12 weeks postfertilization and a SL of 11.2–11.7 mm at the onset of metamorphosis was reported, all values compatible with our own findings.

While emphasis is often put on the morphological and ecological changes that characterize this period,^3^ metamorphosis is also the time of physiological modifications.^34^ Among them, changes in the capacity to osmoregulate at metamorphosis have been reported in several teleost species.^29,30^ Similar metamorphic modifications of osmoregulation are also known in other aquatic groups such as crustaceans.^31,35^ Later in the development of zebrafish, we found that blood osmolality did not change and stayed around 300 mOsm·kg^−1^ in the tested stages, juvenile-adults 2 to 3 months of age and 2-year-old adults.

These ontogenetic changes in the capacity to osmoregulate are related to the development of ionocytes in several organs. After hatching at 2–3 dpf, these Na^+^/K^+^-ATPase-rich cells are first found on the skin of the larva, particularly on the yolk sac^15,23^ and they appear on the gills.^36^ Also from 2–3 dpf, the pronephros is formed and appears functional with evidence of ultrafiltration in glomeruli and abundant Na^+^/K^+^-ATPase in tubule ionocytes.^37^ Along active ion uptake from fresh water in the skin ionocytes that compensates diffusive ion loss, the pronephros most probably participates in osmoregulation by producing dilute urine that eliminates excess water while limiting ion loss.

In the European sea bass *Dicentrarchus labrax*, increased blood osmolality during development^29^ has been correlated to an increase in the number of ionocytes.^38^ In the zebrafish, large numbers of ionocytes also begin to occur on the gills around 5–7 dpf.^23^ Between this time and 14 dpf, the osmoregulatory function through ion uptake from fresh water appears to shift from the skin to the gills.^23^

On the gills, at least five types of ionocytes have been identified expressing different transporter enzymes (such as Na^+^/K^+^-ATPase and V-H^+^-ATPase) and several ion channels; they are involved in Na^+^ uptake/H^+^ secretion/NH_4_^+^ excretion, Ca^++^ uptake, Na^+^/Cl^−^ uptake, K^+^ secretion, and Cl^−^ uptake/HCO3^-^ secretion.^17–19^ In particular, regarding the uptake of one of the major osmoeffector ions from fresh water, physiological evidence points to the role of Na^+^/H^+^ exchanger-, of H^+^-ATPase/ENaC-, and of NCC-mediated Na^+^ uptake. In addition, several hormones, among them prolactin and cortisol, have been shown to regulate ion transport through specific receptors.^19^

The timing of occurrence of these cells and of the different transporters, and of the release of the hormones involved in ion transport is still only partly known,^13,14,17–19^ and future research should be directed at their ontogeny. We thus hypothesize that the increase in body fluids/blood osmolality observed in the present study between 5-day-old larvae and 3-week-old larvae originates from a higher ion uptake efficiency of the gills compared with the skin, and also perhaps from an improved function of the kidney. The structure and functions of zebrafish ionocytes have been mainly studied during early development, in embryos and early larvae.^17–19^ As the osmoregulatory performance increases at metamorphosis, it would be worthwhile to explore their changes during the metamorphic transition.

Slight differences in the surrounding medium osmolality have been shown to induce alteration of the structure of zebrafish organs such as eye lenses.^21^ Blood osmolality has thus to be taken into account for histological or experimental work as cell and tissue culture. We have found that blood osmolality increases by ∼60 mOsm·kg^−1^ through different developmental steps from early larvae to adults. These values can be used to adjust the osmolality of fixative, cell and tissue culture media, etc., according to the studied postembryonic stage.

In conclusion, during the postembryonic development of *D. rerio*, blood osmolality is close to 240 mOsm·kg^−1^ in early larvae. It progressively increases to ∼270 mOsm·kg^−1^ during the larval development before reaching ∼300 mOsm·kg^−1^ after metamorphosis in juveniles, and later in adults. These ontogenetic changes in blood osmolality illustrate the physiological changes in osmoregulation associated with organ development in early larvae and later with metamorphosis. They open to possible further studies on the cellular (ionocytes) and molecular modifications associated with the changes in osmolality at metamorphosis. Finally, these values are of practical interest for adjusting the osmolality of fixatives and cell and tissue culture media for research on the zebrafish model.

## Supplementary Material

Supplemental data
