# Supplementary material for: Ontogenetic Changes in Blood Osmolality During the Postembryonic Development of Zebrafish (Danio rerio)
Source: Zebrafish. 2022 Feb 14;19(1):1–6. doi: 10.1089/zeb.2021.0075 (PMC8884165; doi:10.1089/zeb.2021.0075)
Supplement: Supplemental data [file Suppl_TableS1.docx]

Supplementary Table S1. Statistical analysis of blood osmolality at different stage of *Danio rerio*.

L: Larva; Juv: Juvenile; Ad: Adult; d: day; wk: week; m: month; yr: year.

| **Kruskal-Wallis one-way ANOVA test (p<0,001) with Dunn’s multiple comparisons post-test** | | | |
| --- | --- | --- | --- |
|  | significant |  | p value |
| L 5d vs. L 3wk | No | ns | >0.9999 |
| L 5d vs. L-Juv 6wk >8mm | Yes | ** | 0.0011 |
| L 5d vs. L-Juv 6wk <8mm | No | ns | >0.9999 |
| L 5d vs. Juv-Ad 2m | Yes | *** | 0.0005 |
| L 5d vs. Juv-Ad 3m | Yes | * | 0.0498 |
| L 5d vs. Adult 2yrs | Yes | *** | 0.0003 |
| L 3wk vs. L-Juv 6wk >8mm | Yes | *** | 0.001 |
| L 3wk vs. L-Juv 6wk <8mm | No | ns | >0.9999 |
| L 3wk vs. Juv-Ad 2m | Yes | *** | 0.0007 |
| L 3wk vs. Juv-Ad 3m | No | ns | 0.261 |
| L 3wk vs. Adult 2yrs | Yes | *** | 0.0002 |
| L-Juv 6wk >8mm vs. L-Juv 6wk <8mm | Yes | *** | <0.0001 |
| L-Juv 6wk >8mm vs. Juv-Ad 2m | No | ns | >0.9999 |
| L-Juv 6wk >8mm vs. Juv-Ad 3m | No | ns | >0.9999 |
| L-Juv 6wk >8mm vs. Adult 2yrs | No | ns | >0.9999 |
| L-Juv 6wk <8mm vs. Juv-Ad 2m | Yes | *** | 0.0002 |
| L-Juv 6wk <8mm vs. Juv-Ad 3m | No | ns | 0.2264 |
| L-Juv 6wk <8mm vs. Adult 2yrs | Yes | *** | <0.0001 |
| Juv-Ad 2m vs. Juv-Ad 3m | No | ns | >0.9999 |
| Juv-Ad 2m vs. Adult 2yrs | No | ns | >0.9999 |
| Juv-Ad 3m vs. Adult 2yrs | No | ns | >0.9999 |
